# A secure routing approach based on league championship algorithm for wireless body sensor networks in healthcare

**DOI:** 10.1371/journal.pone.0290119

**Published:** 2023-10-02

**Authors:** Mehdi Hosseinzadeh, Adil Hussein Mohammed, Amir Masoud Rahmani, Farhan A. Alenizi, Seid Miad Zandavi, Efat Yousefpoor, Omed Hassan Ahmed, Mazhar Hussain Malik, Lilia Tightiz

**Affiliations:** 1 Institute of Research and Development, Duy Tan University, Da Nang, Vietnam; 2 School of Medicine and Pharmacy, Duy Tan University, Da Nang, Vietnam; 3 Department of Communication and Computer Engineering, Faculty of Engineering, Cihan University-Erbil, Erbil, Kurdistan Region, Iraq; 4 Future Technology Research Center, National Yunlin University of Science and Technology, Yunlin, Taiwan; 5 Electrical Engineering Department, College of engineering, Prince Sattam Bin Abdulaziz University, Al-Kharj, Saudi Arabia; 6 School of Biotechnology and Biomolecular Science, The University of New South Wales, Sydney, Australia; 7 Department of Computer Engineering, Dezful Branch, Islamic Azad University, Dezful, Iran; 8 Department of Information Technology, University of Human Development, Sulaymaniyah, Iraq; 9 School of Computing and Creative Technologies College of Arts, Technology and Environment (CATE) University of the West of England Frenchay Campus, Bristol, United Kingdom; 10 School of Computing, Gachon University, Seongnam, Korea; TU Wien: Technische Universitat Wien, AUSTRIA

## Abstract

Patients must always communicate with their doctor for checking their health status. In recent years, wireless body sensor networks (WBSNs) has an important contribution in Healthcare. In these applications, energy-efficient and secure routing is really critical because health data of individuals must be forwarded to the destination securely to avoid unauthorized access by malicious nodes. However, biosensors have limited resources, especially energy. Recently, energy-efficient solutions have been proposed. Nevertheless, designing lightweight security mechanisms has not been stated in many schemes. In this paper, we propose a secure routing approach based on the league championship algorithm (LCA) for wireless body sensor networks in healthcare. The purpose of this scheme is to create a tradeoff between energy consumption and security. Our approach involves two important algorithms: routing process and communication security. In the first algorithm, each cluster head node (CH) applies the league championship algorithm to choose the most suitable next-hop CH. The proposed fitness function includes parameters like distance from CHs to the sink node, remaining energy, and link quality. In the second algorithm, we employs a symmetric encryption strategy to build secure connection links within a cluster. Also, we utilize an asymmetric cryptography scheme for forming secure inter-cluster connections. Network simulator version 2 (NS2) is used to implement the proposed approach. The simulation results show that our method is efficient in terms of consumed energy and delay. In addition, our scheme has good throughput, high packet delivery rate, and low packet loss rate.

## 1 Introduction

In recent years, low-energy electrical circuits have been designed to create wireless communication. This has contributed to produce tiny, low-energy, and cheap electronic equipment such as smart sensors, which can be installed on different objects for measuring different factors [1, 2]. There are various sensors such as thermal sensor, magnetic sensor, light sensor, mechanical sensor, and chemical sensor [3, 4]. Wireless sensor networks (WSNs) involve a number of sensor nodes scattered in an environment for monitoring various parameters [5, 6]. They have different applications in many areas, for example battlefield monitoring, environmental monitoring, health monitoring [7, 8], monitoring the irrigation process of agricultural products, and home automation [9]. Today, WSNs are applied to monitor individuals and improve their lives. These networks are known as a promising technology in electronic health [10]. Wireless body sensor network (WBSN) is a subset of WSNs. It is created when a number of biosensor nodes are installed inside or on the human body to measure vital signs and health data such as heart rate, body temperature, and blood glucose and control the human body activities [11, 12]. In a WBSN, a biosensor forms a multi-hop path to transfer data packets to the sink node. Then, the sink node sends all collected data to a central server to store this data. Finally, doctors can analyze the data stored on the central server and remotely decide on the health status of individuals [13, 14]. Note that the application of WBSNs is not limited to healthcare. They have many applications, including entertainment, healthcare, sport, and military, for example, these networks can monitor the health status of patients, control the body when training sport to professional and beginner people, and monitor the sleeping stages. Additionally, they can be used in remote medical systems, entertainment applications, motion detection, secure authentication, and essential services [15–17].

Routing is a challenging issue in WBSNs due to their particular characteristics, including limited resources, unreliable communication links, operation without a supervisor, and lack of central management [18–20]. In addition, biosensors have usually constraints in terms of battery, processing power, and memory, and it is not easy to recharge or replace their battery, especially when they are inside the human body [21–24]. These limited resources affect significantly the routing process in terms of network lifetime, routing overhead, packet loss, and delay [25, 26]. Additionally, these nodes have faced challenges like short transmission range, interference, packet loss, and resource allocation issue. These challenges affect negatively data transmission [27–29]. In fact, due to the unique WBSN characteristics, the existing protocols cannot work properly in these networks, and it is necessary to design an energy-efficient routing approach. In recent years, energy-efficient routing methods have been proposed for WSNs [30–32]. However, these routing protocols cannot be used in WBSNs.

On the other hand, when WBSNs are used in the healthcare applications, they must be monitored continuously to maintain privacy of individuals and timely deliver data packets to the destination. Thus, network security is an essential need for such applications since health data are very critical. If malicious nodes modify the health data, then doctors work on false data and perform a false analysis, which leads to false detection and wrong decisions. Also, patients do not tend to make the health data available to anyone because if adversaries earn the health information, they may misuse this information and damage their personal and social life [24, 27]. In this application, efficient-energy and secure routing approach is an important issue to deliver the health data of individuals securely to the destination and prevent adversarial operations by attackers. Although biosensors have very limited resources. This issue limits the design of complex security mechanisms. Therefore, security components, like data integrity, data confidentiality, authentication, and data availability must be guaranteed in a secure routing according to constraints of resources in WBSN to avoid the weak network performance due to routing attacks. Although, a secure technique cannot meet all security requirements in WBSNs. In recent years, efficient-energy routing solutions have been proposed [30–32]. However, the design of the lightweight security mechanisms in these methods has not been studied.

In this paper, we propose a secure routing method using the league championship algorithm (LCA) for wireless body sensor networks. This method can create a tradeoff between energy consumption and security in the network. In our method, the routing problem is defined as an optimization issue. In this scheme, we use the league championship algorithm (LCA) to find the next-hop node and create energy-efficient energy paths. According to our knowledge, no routing method has used the LCA algorithm so far in WBSN. In addition, this method designs a lightweight encryption mechanism to provide network security. The main contributions in our method are stated as follows:

- In this method, each cluster head node (CH) uses LCA to prioritize their neighbors for sending the route request (RREQ) message. Thus, we propose a fitness function with regard to distance from CHs to the sink node, remaining energy, and link quality. When the neighboring cluster heads are prioritized, the CH prepares the RREQ message and broadcasts it to high-priority CHs. This issue lowers routing overhead, manages network congestion, and distributes the consumed energy between network nodes evenly.
- The proposed scheme uses an encryption technique to secure messages. In each cluster, cluster member nodes (CMs) use symmetric keys to secure intra-cluster communication. This lowers their consumed energy and memory. On the other hand, asymmetric keys provide secure connections between CHs.

Our paper has the following organization: Section 2 expresses the related works. We demonstrate the basic concepts in Section 3. Our system model is described in Section 4. Section 5 demonstrates our scheme in details. In Section 6, we study the security of our method. next, Section 7 analyzes the routing overhead in various routing approaches. Section 8 simulates our scheme and evaluates its results. Ultimately, Section 9 demonstrates our conclusions in this article.

## 2 Related works

In [30], the secure multi-tier energy-efficient routing approach (SMEER) is proposed in heterogeneous wireless sensor networks. SMEER seeks to enhance security and energy saving in the network. This scheme clusters nodes in several groups in accordance with the K-means method. In each group, the nodes employ the ant lion optimization algorithm (ALO) to specify the foremost node as the cluster head node. Clustering makes better performance with regard to energy consumption, network lifetime, and scalability. However, ALO increases the computational and communication overheads. SMEER utilizes an elliptic curve cryptographic (ECC) method to provide security when transferring data packets to the sink node. Although, this key cryptography technique is asymmetric and consumes more energy, but provides further security in the network.

In [31] a safe routing approach based on multi-objective ant colony algorithm (SRPMA) is presented in WSN. In this method, the ant colony algorithm is converted into a multi-objective routing algorithm. This approach regards two goals, trust and residual energy for this optimization issue to achieve an optimal solution, which improves security and lifetime. SRPMA uses D-S evidence theory to design a trust evaluation model. This routing method considers only energy and trust when finding different paths. However, considering other factors, for example, connection quality and distance can improve the routing process. This method considers a flat topology and does not design any clustering technique. This decreases its scalability. Also, this method has a large computational and communication overhead because it utilizes the ant colony algorithm. Therefore, the routing process boosts energy consumption and delay.

In [32], a blockchain and reinforcement learning-based secure routing scheme (RLBC) is offered in WSNs. This scheme consists of two sections, including routing and blockchain network. The task of the blockchain network is to increase trust and stability when exchanging routing information because it makes routing information, traceable and tamper-proof. In the routing process, nodes learn dynamically the best path using reinforcement learning algorithm. The blockchain network records the path information in each hop. Thus, if there are routing loops, invalid links, or low transmission rate, this algorithm does not allow to pass data packets through the paths. This helps RLBC to dynamically select efficient and reliable paths. Although, RLBC experiences a high computational cost and a big time complexity because of reinforcement learning and blockchain. This causes a high-delay routing operation in the network. Furthermore, this scheme does not pay attention to the energy parameter, and its conclusion is that energy is not evenly distributed in the network. Moreover, this approach is not scalable because the clustering process is not considered in RLBC.

In [33], the information-aware secure routing (IASR) is suggested for WSN. In IASR, the Dijkstra method is modified to form secure routes between network nodes. In this scheme, two factors, including status and trust are employed to pick out the next-hop node. For defining the trust system, IASR analyses normal or abnormal behaviors of nodes when transmitting former data packets to earn the attack probability. Also, the status factor is defined in accordance with a combination of residual energy and distance from nodes to the base station. As a result, IASR creates secure paths with a minimum cost. These secure paths can deal with various attacks. IASR is a distributed routing algorithm, which utilizes local information when choosing the next-hop node. Although, the clustering process is not considered in IASR, Thus, it is not scalable. Also, routes may be delayed in IASR.

In [34], the safe and low-energy zone-based routing approach (SeLeZoR) is presented in WSNs. In SeLeZoR, network is partitioned into different zones, and these zones are categorized into a number of unequal clusters. When the distance from clusters to the sink node is long, then the clusters are large. In contrast, when clusters are very near to the sink node, they are small. This issue helps SeLeZoR to enhance scalability, balance energy consumption, and decrease network traffic. Cluster member nodes send their collected data packets with a least transfer power to the cluster head. They use the received signal strength index (RSSI) to determine the minimum transmission power. Cluster heads send the enciphered packets to their zone head. The task of zone head is to forward the packets to the sink node through a safe and appropriate route. In this scheme, a key management mechanism is presented to insure secure connections. However, this mechanism is not properly introduced. This scheme utilizes the time division multiple access (TDMA) to employ the transfer channel. However, this method has a low security level because it applies only symmetric key cryptography.

In [35], the ad hoc on-demand distance vector (AODV) is proposed in mobile ad hoc networks. In this approach, network nodes adjust routing tables to store the information about the suitable node for reaching the desired destination. When two nodes (source and destination) want to connect to each other and they do not have a valid route in their routing table, they must discover a valid route between themselves. In this process, it utilizes the route request packets (RREQs) and the route reply packets (RREPs). AODV inserts a sequence number in RREQs to guarantee that these packets are fresh and the created paths are free-loop. AODV introduces a route maintenance mechanism for detecting and repairing the failed routes. However, AODV experiences a high delay in the routing operation when the size of the network is large.

In [36], the centralized low energy adaptive clustering hierarchy scheme (LEACH-C) is introduced. LEACH-C introduces a centralized clustering scheme based on the simulated annealing algorithm for forming clusters. LEACH-C seeks to create better clusters, which balance consumed energy between network nodes. In this clustering scheme, nodes send two parameters, including the current position and their energy status to the sink node, which is responsible for performing the clustering process and dividing sensor nodes in several groups. In the clustering process, the base station computes the average network energy and compares the energy of each node with the average value. The base station removes nodes whose energy is fewer than the average value from the CH-candidate set. Then, the base station executes the simulated annealing algorithm to select the most suitable CHs from the CH-candidate set. This algorithm considers the minimum sum of the squared distances between cluster members and CHs to lower energy used by CMs when transferring data to the corresponding cluster head. However, this algorithm is not consistent with WSNs because in hostile environments, the centralized clustering algorithm suffers from the single point of failure problem.

In [37], the dynamic rate aware classified key distributional secure routing (DRCKDS) is suggested in WSNs. In DRCKDS, packets are categorized according to their sensitivity, and nodes are divided with regard to their importance. Next, DRCKDS utilizes these categorizations for distributing safe keys. This idea reduces energy consumption since low-importance data has less security. In this routing scheme, each node utilizes a neighboring table to discover the new paths. However, the routing process is not explained properly. This table stores the neighbors’ information, for example type, position, and the status of transmissions and re-transmissions. Finally, DRCKDS evaluates paths based on the secure route measure (SRM), which is achieved in accordance with the behavior of the nodes (i.e. transmissions and re-transmissions) in the path. Although, it is not clear how to analyze trust of nodes. Eventually, DRCKDS uses symmetric keys to make a secure data transfer operation.

In [38], a tree-based secure routing method by means of a dragonfly algorithm called CTSRD is introduced for Internet of Things. CTSRD has a weighted trust system (W-Trust) that is distributed and lightweight and attempts to achieve the trust values of IoT devices. This mechanism punishes trust levels related to attacker nodes by calculating a penalty factor so that these nodes are isolated in the network. Also, the trust levels of normal IoT devices have increased based on an award factor. In addition, a trusted clustering scheme (T-Clustering) is suggested in CTSRD, where cluster head nodes (CHs) are selected from trusted IoT nodes. Further, CTSRD organizes CHs in a routing tree named DA-Tree by means of a dragonfly algorithm (DA). Also, a new objective function has been introduced to calculate the quality of DA-Tree. This tree is secure and permanent. It makes a balanced energy consumption between IoT devices and increases network lifetime. The simulation results exhibit that CTSRD has a better performance compared to other methods (EEMSR and E-BEENISH). Although, the packet delivery rate (PDR) in this scheme is slightly lower than EEMSR.

In [39], the authors focused on mobile WSNs, which include mobile sensors with a fixed velocity, and introduced two challenging issues in these networks, namely energy saving and data availability. Then, they offered an energy-aware and data availability-based routing approach called REDAA in WSNs. REDAA attempts to find the most stable paths and best cluster heads because it seeks to improve the network lifetime. This scheme integrates two clustering methods, namely Q-LEACH and MH-LEACH to form clusters in the network. Then, communication routes are created between these cluster heads. These paths guarantee data availability. Finally, energy saving is guaranteed in the data collecting process because it focuses on slot-based code division multiple access techniques. The evaluations performed in this paper show that REDAA improves throughput and energy consumption.

In [40], the authors emphasized the importance and necessity of routing and secure data transmission because they prevent attackers to access health information illegally. Then, a secure routing approach named SecAODV is suggested for heterogeneous WBSNs. This scheme defines three components, namely the bootstrap component, the routing component, and the security component. The bootstrap component is run by the base station to put the related commands and functions in the storage of nodes. The routing component defines how to decide on next-hop nodes based on the score calculated for each cluster head. Four parameters, including current energy, distance, hop count, and link quality are combined with each other to obtain this score. The security component explains the cryptography process in the network so that cluster members use a symmetric key to protect their data, but cluster heads apply an asymmetric key to encrypt their data. This scheme is evaluated and the results prove that SecAODV decreases delay and consumed energy and increases PDR and throughput in the network.

According to the methods studied in this paper, we can find that in recent years energy-efficient routing solutions, for example SMEER [30], SeLeZoR [33], and LEACH-C [36] have been proposed. However, these methods do not succeed in designing efficient and lightweight security mechanisms. In some of the routing techniques such as AODV [35] and LEACH-C [36] any proper security mechanisms are not designed. Also, poor security mechanisms are designed in other methods such as SeLeZoR [33] and DRCKDS [37]. This has limited the use of these methods for sensitive applications such as healthcare. On the other hand, some methods, such as RLBC [32], use a complex security mechanism and ignore the limited resources of sensor networks, especially energy. Among these methods, only some of them, such as SRPMA [31] and LASR [33], seek to create a tradeoff between energy and security in the network. This shows that there is a research gap in the field of energy-efficient secure routing techniques. For this reason, we propose a secure routing approach with regard to the league championship algorithm (LCA) for WBSNs. This method attempts to create a tradeoff between energy consumption and security. In this method, we use LCA to choose the next-hop node and obtain energy-efficient paths. In addition, we try to design a lightweight encryption technique to secure network communications. Table 1 expresses the benefits and shortcomings of the methods studied in this section in summary.

**Table 1:** Advantages and disadvantages of related works.

Scheme	Advantages	Disadvantages
SMEER [30]	Scalability, network clustering, energy efficiency, improving network security	High computational and communication overhead due to the use of asymmetric encryption technique and ALO
SRPMA [31]	Creating a tradeoff between security and energy efficiency in the routing process using the ant colony algorithm, designing a security model based on the evaluation of trust of nodes	Ignoring connection quality, distance, and traffic when designing optimal paths, high computational overhead, and high communication overhead
RLBC [32]	Designing a secure and trusted routing technique, acceptable security level, considering the data transmission rate when finding paths, and avoiding the choice of paths with high congestion	Designing a complex security mechanism based on blockchain, computational complexity, high communication overhead, ignoring the limited energy source in the routing process
IASR [33]	Creating a tradeoff between energy consumption and security, designing a security model based on trust assessment, regarding energy in the routing operation	Not considering link quality when selecting paths, creating unstable paths, long delay for discovering paths
SeLeZoR [34]	Network clustering, creating unequal clusters, balancing energy consumption in the network, reducing traffic in the network	Designing a weak security mechanism
AODV [35]	Designing an on-demand routing method, creating loop-free paths	Not considering energy efficiency, high latency and high bandwidth consumption in the routing process, not designing a security mechanism
LEACH-C [36]	Clustering, scalability, balancing energy consumption in the network	Single point of failure issue, not designing a lightweight security mechanism
DRCKDS [37]	Considering different security levels in the network, allocating high security level for sensitive data and low security level for low-sensitive data	Not defining the routing process, not explaining the trust evaluation process, using symmetric encryption to secure the network
CTSRD [38]	An energy-aware powerful routing, uniform distribution of energy between nodes, reducing energy consumption, increasing network lifetime, designing a strong defense mechanism against attacks and isolating malicious nodes	Reducing the packet delivery rate (PDR)
REDAA [39]	Considering energy saving and data availability, forming the most stable paths in the network, finding best cluster heads, improving network lifetime	Not designing a security mechanism
SecAODV [40]	Clustering, creating a trade-off between energy efficiency and network security, Considering the residual energy of node when finding new paths, the use of symmeric and asymmetric keys to secure communication links	High routing overhead, high latency in the routing process

## 3 Basic concepts

In this section, we express the league championship algorithm (LCA) due to its application in the proposed scheme. In recent decades, meta-heuristic algorithms have been widely used to solve networking problems, especially routing and clustering in wireless sensor networks. According to [41], the routing process for finding the best paths in the network is a NP-complete problem. Therefore, it is very difficult and time-consuming to solve the routing problem, especially in large-scale networks, to achieve the best route between two nodes. To solve such a routing problem, an effective solution is to use meta-heuristic algorithms to find near-to-optimal responses. For example, in [41, 42], the authors describe how to use meta-heuristic algorithms to solve the routing problem in wireless sensor networks. Today, the League Championship Algorithm (LCA) has gained popularity among researchers in different research fields because of its potential and ability to solve real-world optimization problems. LCA has a great ability to solve optimization problems because it can find near-to-optimal responses at a high convergence speed. In [43], this algorithm has been tested in different areas and has proven its ability compared to other optimization methods. Therefore, in this paper, LCA is used to improve the routing process.

### 3.1 League Championship Algorithm (LCA)

The league championship algorithm (LCA) is a meta-heuristic technique, which can solve continuous optimization issues [43]. LCA produces high-quality solutions at higher convergence speed than other techniques, for example, genetic algorithm (GA) and particle swarm optimization (PSO). LCA follows the following rules [43]:

- There is more likely that the team having more gaming strength wins the game.
- It is not possible to fully predict the game result based on the gaming strength of teams.
- There is the same probability that team *i* defeats team *j* in a game in the view of both teams.
- According to game results, teams are only winner or loser. This means that this algorithm does not consider the tie status.
- When team *i* defeats team *j* in a game, any strength point that has led to team *i* to win, is regarded as a weakness point for team *j* that has led to its failure.
- Setting the structure of each team is determined only based on last week’s events.

In LCA, each team structure (response) can be displayed as an 1×*n* vector of real numbers, which *n* is the number of variables in the desired problem. Each element of the vector is considered as a player, which indicates the corresponding variable value. Each change in the corresponding variable means that the player changes in the team structure. *f*(*X* = (*x*_1_, *x*_2_, …, *x*_*n*_)) is an objective function with *n* variable, which must be optimized in the search environment. A team structure (i.e. possible response) for team *i* at week *t* can be expressed as $X_{i}^{t}=(x_{i,1}^{t},x_{i,2}^{t},\ldots ,x_{i,n}^{t})$. Each team (team *i*) stores its best team structure ($B_{i}^{t}=(b_{i,1}^{t},b_{i,2}^{t},\ldots ,b_{i,n}^{t})$) until week *t*.

In this algorithm, the league represents the initial population. In the step one, *L* solutions are randomly formed. Next, the solutions are gradually convergent to the optimal result. This algorithm ends after *S* seasons (the stop condition of the algorithm) and the winner team is selected as the final response. Each season involves *L* − 1 weeks, and each week includes *L* × (*L* − 1)/2 games so that *L*/2 games are held parallel. Therefore, the number of iterations (steps of algorithm) is considered as *S* × (*L* − 1) weeks. LCA consists of three main steps [43]:

**Generating the league schedule** This step specifies the time of games in a season. To specify the league schedule, the single round robin technique is used.

**Determining winner or loser** In each weak, teams play with each other, the result can be win or lose. The results are determined according to gaming strength of teams (i.e. objective function). Team *i* calculates its win probability to overcome team *j* at week *t* through Eq 1:

$$\begin{array}{c} p_{i}^{t}=\frac{f(x_{j}^{t})-\hat{f}}{f(x_{i}^{t})+f(x_{j}^{t})-2\hat{f}} \end{array}$$ (1)

where $p_{i}^{t}$ indicates the win probability of team *i* when playing with team *j* at week *t*. $f(x_{i}^{t})$ and $f(x_{j}^{t})$ are the gaming strength of teams *i* and *j*, respectively. $\hat{f}$ is the best explored value function so far; it is equal to $\hat{f}=\underset{i=1,\ldots ,L}{\text{min}}\;\{f(B_{i}^{t})\}$. Also, according to the rules of LCA, we have:

$$\begin{array}{c} p_{i}^{t}+p_{j}^{t}=1 \end{array}$$ (2)

After calculating the chances, the random number, *r* is produced so that *r* ∈ (0, 1). If $r\leq p_{i}^{t}$, then team *i* is a winner team at week *t*. Otherwise, team *j* is a winner team at week *t*.

**Creating a new team structure** To enhance the team’s performance at later week, coaches evaluate internal and external scales. For example, some internal scales are weaknesses and strengths in the team and players, and some external scales are opportunities and threats of the opposite team to form the new team structure. This new structure is calculated based on strengths/weaknesses/opportunities/threats (SWOT) analysis. For more details, see [43].

## 4 System model

**Fig 1:**
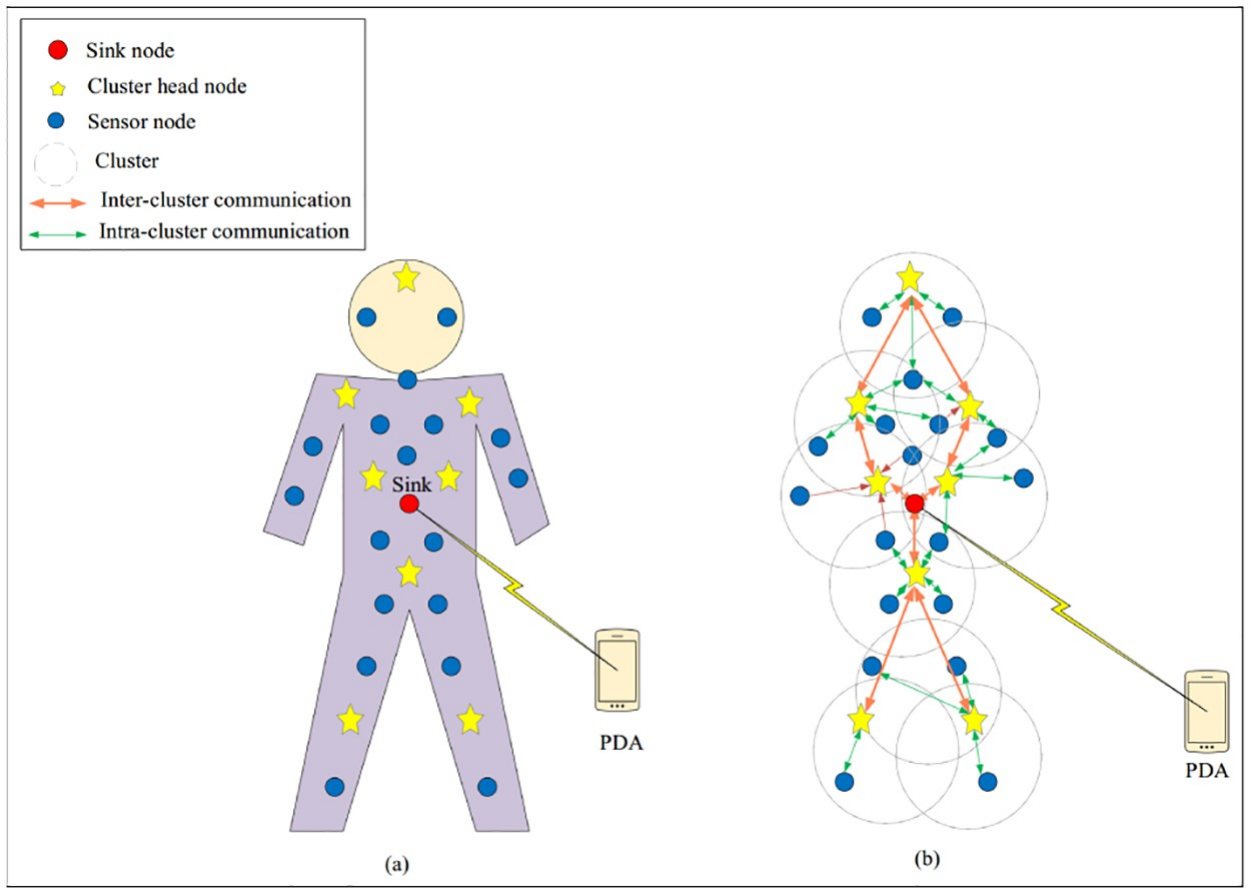
Network model in proposed method (a) location of biosensors on body (b) communication between nodes.

The proposed approach regards a heterogeneous WBSN that includes the number of biosensors. We assume that the network is clustered using the low energy adaptive clustering hierarchy (LEACH) algorithm [44]. This network model includes various cluster head nodes (CHs) and cluster member nodes (CMs), and a sink node. The network supports several communications, including CH-to-CH, CH-to-CM, CM-to-CH, CM-to-CM connections. As shown in Fig 1, the sink node is located in the middle of the human body. In the following, we explain the tasks of each biosensor and its communications.

- **Cluster member nodes**: These biosensors are responsible for monitoring human vital signs such as blood glucose, blood pressure, body temperature, electrocardiogram (ECG), electroencephalogram (EEG), and electromyogram (EMG). In Fig 1(a), these biosensors are marked with blue color. According to Fig 1(b), these nodes can directly communicate with CHs.
- **Cluster head nodes**: The task of these biosensors is to collect data from CMs, and transmit it to the sink node. In Fig 1(a), these nodes are shown in star form. These biosensors communicate with the sink node via a multi-hop path, which is also represented in Fig 1(b).
- **Sink node**: Task of this node is to receive data from CHs and transmit it to a control center such as a personal digital assistant (PDA) or smartphone. The sink node is shown with red color in Fig 1(a) and is located in the middle of the human body.

### 4.1 Attack model

Attackers are trying to make different attacks and damage network performance. Our scheme guarantees data confidentiality in the network. Data confidentiality ensures that sensitive information is protected in the network and unauthorized users cannot obtain this information [45, 46]. Therefore, our scheme protects the network against eavesdropping and traffic analysis attacks. In this case, the attacker can be internal or external. We assume that attackers do not inject any fake packets in the network, and they cannot disrupt the routing process. This means that these attackers are passive. Thus, malicious nodes employ information or hear communication channels without affecting network performance. In our method, the following hypotheses are considered for attackers:

- An attacker can eavesdrop on all connections and access data transferred on wireless links.
- An attacker can capture nodes and reach their secret information like encryption keys, identifier, and important data.
- An attacker tries to abuse the obtained data for compromising other nodes in the network.

## 5 Proposed method

Our scheme involves three parts explained in the following subsections:

- Bootstrapping step
- Routing step
- Communication security step

### 5.1 Bootstrapping step

Before the network is launched, the sink node assigns a certain ID and a specific key (*k*_*i*,*BS*_) to each biosensor. Also, it regards an initial key (*k*_*initial*_) for nodes. This key is known by all nodes, and its goal is to make secure wireless links. The sink node freshens this key periodically or when capturing a node in the network. Then, the sink node ciphers this new key by *k*_*i*,*BS*_ and unicasts it for valid nodes in the network. As a result, captured or dead nodes cannot access this key. Also, the sink node loads several encryption factors in the memory of CHs. These factors are used to secure the wireless links between biosensors in the network (i.e. communication between CH and CMs and communication between CHs). These factors are:

- **A key source**: It is applied to generate cluster key for securing intra-cluster communication.
- **A pair of public-private keys**: These keys are used for securing communication between CHs.

Our scheme utilizes a lightweight security algorithm because intra-cluster communications are secured by a symmetric key cryptography, which requires less energy than asymmetric cryptography. This helps the cluster member nodes to consume less energy. Also, CMs store only one cluster key in their memory. This reduces memory overhead and routing overhead when providing the intra-cluster security. On the other hand, cluster head nodes use an asymmetric cryptosystem called the elliptic curve cryptographic (ECC) for securing their communication. This encryption technique can provide better security than the Rivest-Shamir-Adleman cryptosystem (RSA) because this method enhances network efficiency by lowering the key size and decreasing consumed energy and creates a suitable security level. CHs store the cluster key, their public-private keys, and the public keys of other CHs. This means that they consume more energy for providing security in the inter-cluster communications. They have higher routing overhead and memory overhead. This hybrid cryptography scheme provides an acceptable security for network connections and consumes energy efficiently [47, 48].

### 5.2 Routing step

In the routing process, each cluster member forwards its data directly (single-hop) to its CH. Next, CH aggregates the data obtained from its cluster members and forwards the combined data to the sink node using a multi-hop manner. Suppose *CH*_*i*_ wants to forward its data packets to the sink node and does not have a path to it. In this case, *CH*_*i*_ begins a route discovery process based on the league championship algorithm (LCA). In the routing process, first, each CH node exchanges a hello message periodically with their neighbors. This message contains the location and remaining energy of each node. After receiving the message, CHs store this information in a neighborhood table to use them in the routing process. Then, *CH*_*i*_ prioritizes the neighboring nodes using LCA to select the most suitable next-hop node. In this process, the structure of each team is displayed as an *N*_*i*_-dimensional vector. So that *N*_*i*_ is the number of single-hop neighboring CHs of *CH*_*i*_. For example, the structure of team *p* at week *t* can be expressed as $Team_{p}^{t}=(Prio_{p,1}^{t},Prio_{p,2}^{t},\ldots ,Prio_{p,N_{i}}^{t})$. In team *p*, each player represents the priority of the corresponding neighboring CH, for example, $Prio_{p,1}^{t}$ corresponds to the priority of the first neighboring CH. Note that each player has a value in the range [0, 1]. When the value of the player is close to one, this means that the corresponding CH has a higher priority. When prioritizing the neighboring nodes, we assume that there are six teams (*L* = 6) in the league (initial population). Also, this algorithm ends after *S* seasons. In the proposed method, *S* = 10. As a result, the algorithm is repeated *S* × (*L* − 1) = 10 × 5 = 50 weeks. Our algorithm includes the following steps:

**1**. The team scales, including the league size (*L* = 6), the number of seasons (*S* = 10), and other control parameters are initialized.

**2**. In this step, each team structure (For example, $Team_{p}^{t}=(Prio_{p,1}^{t},Prio_{p,2}^{t},\ldots ,Prio_{p,N_{i}}^{t}),\;\;\;\;\;p=1,2,\ldots ,6$) is randomly initialized, so that:

$$\begin{array}{c} 0\leq Prio_{p,k}^{t}\leq 1,\;\;\;\;p=1,2,\ldots ,6\;and\;\;k=1,..,N_{i} \end{array}$$ (3)

**3**. League scheduling is determined using the single round robin technique.

**4**. The gaming strength of each team (for example, team *p*) is calculated based on three parameters:

**Distance from the neighbors of *CH*_*i*_ to the sink node**: *CH*_*i*_ prefers to choose the neighbor as its next hop, which is closer to the sink than other neighbors. This leads to fewer hops in the routing paths, and data packets reach the destination at a lower time. So, in a team, if CHs close to the sink node have higher priorities, this team achieves a high gaming strength. The distance from a neighboring CH (such as *CH*_*r*_) to the sink node is calculated according to Eq 4:

$$\begin{array}{c} D_{r}=\sqrt{{\left(x_{r}-x_{Sink}\right)}^{2}+{\left(y_{r}-y_{Sink}\right)}^{2}} \end{array}$$ (4)

so that (*x*_*r*_, *y*_*r*_) and (*x*_*Sink*_, *y*_*Sink*_) are the spatial coordinates of *CH*_*r*_ and the sink node, respectively.

**Remaining energy of the neighboring CHs**: Energy is a highly effective parameter on the stability of paths because if the intermediate nodes in a path have sufficient energy, this path can be used for a longer period of time, and disconnections will be reduced in this path. On the other hand, when the participation of low-energy nodes is reduced in the formation of paths, these nodes can store more energy. As a result, the network lifetime will be improved. In addition, the stability of paths will have a positive effect on reducing the number of lost packets. Thus, it can reduce the need for data retransmission. So, in a team, if high-energy CHs get higher priorities, this team obtains a high gaming strength. Note that CHs know its energy level at any moment. *E*_*r*_ indicates the remaining energy of *CH*_*r*_.

**Quality of links between *CH*_*i*_ and its neighbors** *CH*_*i*_ prefers to select the neighbor as the next hop that has a high-quality link because the quality of the link is effective in determining the stability of paths. Routes that have high-quality links can be used for longer period of time. They decrease the number of lost packets. While routes with low-quality links are broken quickly and lose a large number of data packets, they need to be modified and reconstructed, which is a time-consuming process. So, in a team, if CHs with high-quality links get higher priorities, this team achieves a high gaming strength. The quality of connections in a path is specified with regard to the received signal strength indication (RSSI) [49, 50]. It is a register located in transceivers and calculates the signal strength of the received packets. In [51] asserts that further RSSI can improve the packet reception ratio. In addition, the indicator is fixed for a small interval (for example, two seconds) (with standard deviation less than 1dBm). Therefore, it can approximate connection quality. The quality of link between *CH*_*i*_ and *CH*_*r*_ is shown as *Q*_*r*,*i*_.

Finally, $f(Team_{p}^{t})$ is calculated according to Eq 5:

$$\begin{array}{c} f(Team_{p}^{t})=\sum_{r=1}^{N_{i}}W_{r}[(\frac{Q_{r,i}-q_{\text{min}}}{q_{\text{max}}-q_{\text{min}}})+\left(\frac{E_{r}-e_{\text{min}}}{e_{\text{max}}-e_{\text{min}}})+(1-\frac{D_{r}}{d_{\text{max}}})\right] \end{array}$$ (5)

where *W*_*r*_ is the weight of the neighboring CH (such as *CH*_*r*_). It is determined based on its priority (*ω*_*r*_). When in team *p*, a player (such as *CH*_*r*_) has the highest value, it has the highest priority (*ω*_*r*_ = 1). Note that if two players have same value, the player with smaller index has higher priority. The weight of *CH*_*r*_ is determined based on Eq 6:

$$\begin{array}{c} W_{r}=\frac{N_{i}-(\omega_{r}-1)}{\sum_{i=1}^{N_{i}}i},\;\;r=1,\ldots ,N_{i} \end{array}$$ (6)

According to [49], when RSSI has bigger value, the quality of the corresponding link is better. In this case, PDR is increased. Therefore, we consider *q*_max_ = *RSSI* = 87*dBm* because *PDR* = 99% in this case. Also, we assume that *q*_min_ = *RSSI* = 0*dBm* because *PDR* = 0 in this case. For more details, see [49]. *e*_max_ indicates the highest residual energy of neighboring nodes, which is obtained from the neighborhood table. *e*_min_ is the lowest remaining energy of neighbors. *d*_max_ depends on the network size; for example when the network size is *n*×*m*, then $d_{\text{max}}=\sqrt{n^{2}+m^{2}}$.

**5**. Teams play with each other according to the league schedule to specify the winner or loser. According to LCA, the win probability of team *p* against team *q* in week *t* is achieved using Eq 7:

$$\begin{array}{c} P_{p}^{t}=\frac{f(Team_{q}^{t})-\hat{f}}{f(Team_{p}^{t})+f(Team_{q}^{t})-2\hat{f}} \end{array}$$ (7)

where $f(Team_{p}^{t})$ and $f(Team_{q}^{t})$ are the gaming strength of team *p* and team *q* in week *t*, respectively. $\hat{f}$ indicates the optimal function value (i.e. $\hat{f}=\underset{i=1,\ldots ,L}{\text{min}}\;\{f(B_{i}^{t})\}$). Note that, the following condition is based on the rules of LCA:

$$\begin{array}{c} P_{p}^{t}+P_{q}^{t}=1 \end{array}$$ (8)

After calculating the win probabilities, a random number *r* is produced so that *r* ∈ (0, 1). If $r\leq p_{p}^{t}$, then team *p* will win at week *t*. Otherwise, team *q* wins at week *t*.

**6**. The new team structure is calculated at week *t* + 1.

**7**. If the end condition (i.e. *t* ≥ *S* × (*L* − 1)) is met, the algorithm ends. Otherwise, if the season has ended, go to Step 3. Else, go to Step 4.

**Fig 2:**
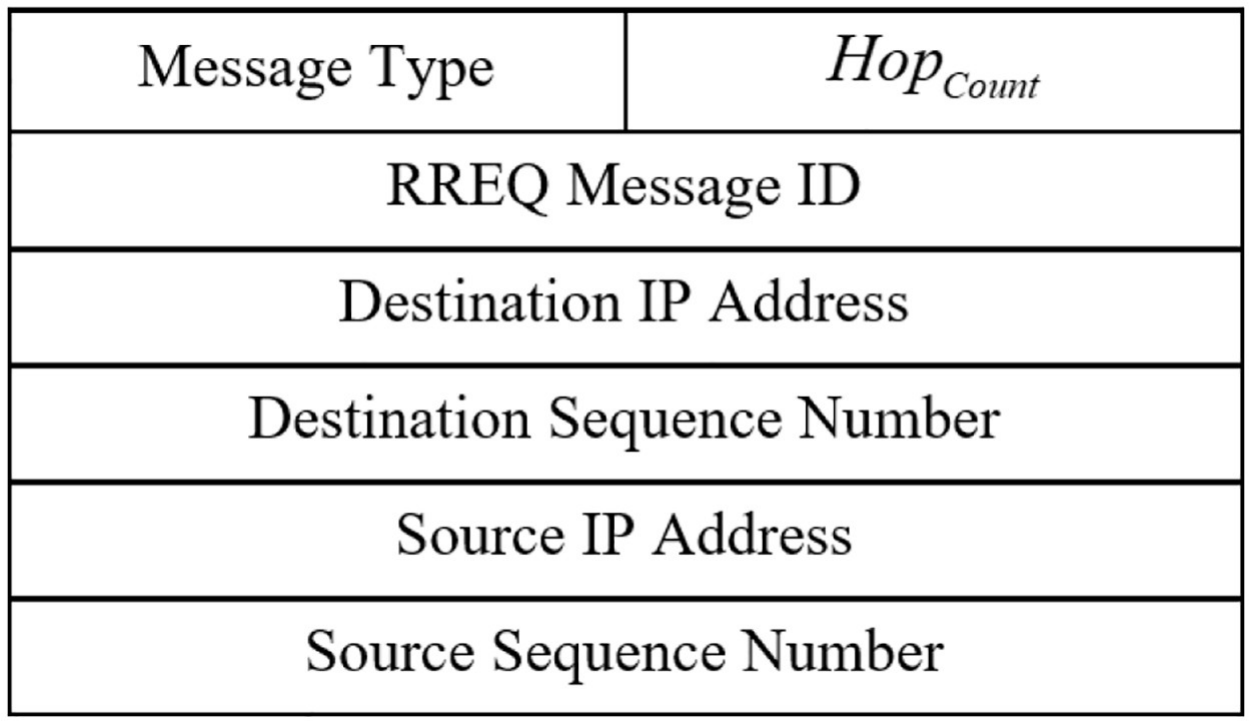
Route request format [35].

After prioritizing the neighboring CHs, *CH*_*i*_ prepares a RREQ packet and sends it to the half of its neighboring CHs with the best priority. The structure of the route request (RREQ) is presented in Fig 2. According to the figure, RREQ format in our method is similar to that in the AODV protocol [35]. Before re-transferring the RREQ packet, *CH*_*r*_ updates the number of hops (i.e. *Hop*_*Count*_ field). This operation continues until RREQ is received by destination.

After RREQ is received by destination, then the destination node prepares a route reply packet (RREP) and transfers RREP to the source CH via the discovered route. After RREP is received by the source CH, this route is recorded in its routing table. The CH transfers its data to destination via this route. Algorithm 1 presents the route discovery algorithm in our method. Note that our scheme uses a path maintenance process similar to AODV. This process checks the validity of the formed routes and repair the failed routes.

Now, we present an analysis of the time complexity related to Algorithm 1. It involves a *For* loop (lines 1–4) and a *While* loop (lines 5–24). As a result, the time complexity depends on these loops:

$$\begin{array}{c} T(n)=T_{For\;\;loop}(n)+T_{While\;\;loop}(n) \end{array}$$ (9)

*For* loop is repeated *N*_*CH*_ times, so that *N*_*CH*_ is the number of CHs in the network. This loop includes two commands (lines 2 and 3), which are executed at fixed times *r*_1_ and *r*_2_, respectively. As a result, *T*_*For loop*_ (*n*) is obtained from Eq 10:

$$\begin{array}{c} T_{For\;\;loop}(n)=N_{CH}(r_{1}+r_{2}) \end{array}$$ (10)

A fixed number *r* ≥ *r*_1_ + *r*_2_ is considered and as a result:

$$\begin{array}{c} T_{For\;\;loop}(n)=N_{CH}(r_{1}+r_{2})\leq N_{CH}\left(r\right) \end{array}$$ (11)

Then, the time complexity corresponding to the *For* loop is *O*(*N*_*CH*_).

Moreover, the *While* loop (lines 5–24) is repeated *N*_*CH*_ times at the worst case. Inside this loop, there are four commands (lines 6–9) and a *While* loop (lines 10–21) and two commands (lines 22 and 23). As a result, *T*_*While loop*_ (*n*) in Eq 12 is equal to:

$$\begin{array}{c} T_{While\;\;loop}(n)=\\ N_{CH}(T_{Four\;\;commands}(n)+T_{While\;\;loop2}(n)+T_{Two\;\;commands}(n)) \end{array}$$ (12)

*T*_*Four commands*_ (*n*) and *T*_*Two commands*_ (*n*) are executed at a fixed times. As a result, they are as follows:

$$\begin{array}{c} T_{Four\;\;commands}(n)\in O(1) \end{array}$$ (13)

$$\begin{array}{c} T_{Two\;\;commands}(n)\in O(1) \end{array}$$ (14)

For calculating *T*_*While loop*2_ (*n*), we know that *While* loop (lines 10–21) is repeated *S* × (*L* − 1) times. It consists of three commands (lines 11, 12, and 13), a *For* loop (lines 14–17), and an *IF* command (lines 18–20). Thus, *T*_*While loop*2_ (*n*) is calculated using Eq 15:

$$\begin{array}{c} T_{While\;\;loop2}(n)=\\ S\times (L-1)(T_{Three\;\;commands}(n)+T_{For\;\;loop2}(n)+T_{IF}(n)) \end{array}$$ (15)

Lines 11, 12 and 13 depend on Eqs 5–8, which is dependent on $N_{Neighbor_{i}}$ (Note that $N_{Neighbor_{i}}$ is equal to *N*_*CH*_ at the worst case). Thus,

$$\begin{array}{c} T_{Three\;\;commands}(n)\in O(N_{CH}) \end{array}$$ (16)

*T*_*For loop*2_ (*n*) is repeated *L* times and *T*_*For loop*2_ (*n*) ∈ *O*(*L*).

Finally, *T*_*IF*_ (*n*) is executed at fixed times (i.e. *T*_*IF*_ (*n*) ∈ *O*(1)). Therefore, Eq 15 is rewritten as Eq 17:

$$\begin{array}{c} T_{While\;\;loop2}(n)=S\times (L-1)\times (m_{1}N_{CH}+m_{2}L+m_{3}) \end{array}$$ (17)

where *m*_1_, *m*_2_, *m*_3_ are constant values.

Now, Eq 12 is equal to:

$$\begin{array}{c} T_{While\;\;loop}(n)=N_{CH}(z_{1}+z_{2}S\times (L-1)\times (m_{1}N_{CH}+m_{2}L+m_{3})+z_{3}) \end{array}$$ (18)

Where *z*_1_, *z*_2_, *z*_3_ are constant values.

If *N*_*CH*_ > *L*, then $T_{While\;\;loop}(n)\in O(SLN_{CH}^{2})$.

Finally, the time complexity of Algorithm 1 in Eq 9 is achieved with regard to Eq 19:

$$\begin{array}{c} T(n)=O(N_{CH})+O(SLN_{CH}^{2}) \end{array}$$ (19)

Thus, $T(n)\in O(SLN_{CH}^{2})$.

**Algorithm 1** Route discovery process

**Input**: *CH*_*source*_: Source node

 *N*_*i*_: The number of single-hop neighboring CHs of *CH*_*i*_.

 *CH*_*k*_: Cluster head nodes (*k* = 1, …, *N*_*CH*_).

**Output**: *Route*_*i*_ between *CH*_*source*_ and *CH*_*destination*_

 **Begin**

1: **for** *k* = 1 to *N*_*CH*_ **do**

2:  **CH**_**k**_: Exchange a *hello message* periodically with their neighbors;

3:  **CH**_**k**_: Store this information in its neighborhood table;

4: **end for**

5: **while** *CH*_*i*_ = *CH*_*destination*_ **do**

6:  **CH**_**i**_: Initialize the team parameters, including the league size, the number of seasons, and other parameters;

7:  **CH**_**i**_: Initialize each team structure as $Team_{p}^{t}=(Prio_{p,1}^{t},Prio_{p,2}^{t},\ldots ,Prio_{p,N_{i}}^{t}),\;\;\;\;\;p=1,2,\ldots ,6$;

8:  **CH**_**i**_: Determine league scheduling according to the single round robin technique;

9:  **CH**_**i**_: Set *t* = 1;

10:  **while** *t* ≥ *S* × (*L* − 1) **do**

11:   **CH**_**i**_: Calculate the gaming strength of each team based on Eq 5;

12:   **CH**_**i**_: Determine $B_{i}^{t}=(b_{i,1}^{t},b_{i,2}^{t},\ldots ,b_{i,n}^{t})$ and $\hat{f}=\underset{i=1,\ldots ,L}{\text{min}}\;\{f(B_{i}^{t})\}$;

13:   **CH**_**i**_: Determine the winner or loser based on Eq 7;

14:   **for** *k* = 1 to *L* **do**

15:    **CH**_**i**_: Calculate the new team structure;

16:    **CH**_**i**_: Update $B_{i}^{t}=(b_{i,1}^{t},b_{i,2}^{t},\ldots ,b_{i,n}^{t})$;

17:   **end for**

18:   **if** *mode*(*t*, *L* − 1) = 0 **then**

19:    **CH**_**i**_: Determine league scheduling according to the single round robin technique;

20:   **end if**

21:  **end while**

22:  **CH**_**i**_: Send *RREQ message* to the half of its neighboring CHs with the best priority;

23:  **Neighboring CH**: Set itself as *CH*_*i*_;

24: **end while**

25: **CH**_**destination**_: Send back *RREP message* to *CH*_*source*_;

26: **CH**_**source**_: Insert the information of this path into its routing table;

 **End**

### 5.3 Communication security phase

In this phase, the intra-cluster security process and the inter-cluster security process are described in details.

#### 5.3.1 Intra-cluster security process

In our scheme, a symmetric key cryptography approach named RC4 secures communication links within cluster. In 1984, Ronald Rivest introduced a well-known stream cipher called Rivest Cipher 4 (RC4), which is widely employed in many networking protocols because of its high speed, simpleness, and easy execution [52]. In this byte-oriented stream cipher, an 8-bit ciphertext is produced by executing the XOR operator on an 8-bit plaintext and an 8-bit key. The size of this secret key obtained from the one-byte keys in the key stream, can be between 1 and 256 bytes. Researchers believed that the security of RC4 is guaranteed when the 16-byte or more secret keys are produced by this algorithm and smaller key sizes are not secure. For more details, refer to [52]. Symmetric cryptosystems are efficient in terms of energy. This helps CMs to consume less energy. The task of cluster head node (like, *CH*_*i*_) is to produce the cluster key (*k*_*cluster*_) and transfer this key to its CMs (like, *CM*_*j*_). When clusters are created, and each cluster head is inform of its members, *CH*_*i*_ randomly picks out a key from the key source. Not that the key source is stored in the memory of *CH*_*i*_ in the bootstrapping phase. Now, *CH*_*i*_ ciphers *k*_*cluster*_ by *k*_*initial*_ and sends the encrypted key to *CM*_*j*_. Eq 20 describes the step.

$$\begin{array}{c} CH_{i}\to *:Encrypt_{k_{initial}}(k_{cluster},ID_{CH_{i}}) \end{array}$$ (20)

After receiving this encrypted message, *CM*_*j*_ deciphers it using *k*_*initial*_. Then, *CM*_*j*_ must check the ID inserted into the message. Finally, it obtains *k*_*cluster*_ based on Eq 21.

$$\begin{array}{c} CM:Decrypt_{k_{initial}}(k_{cluster},ID_{CH_{i}}) \end{array}$$ (21)

**Fig 3:**
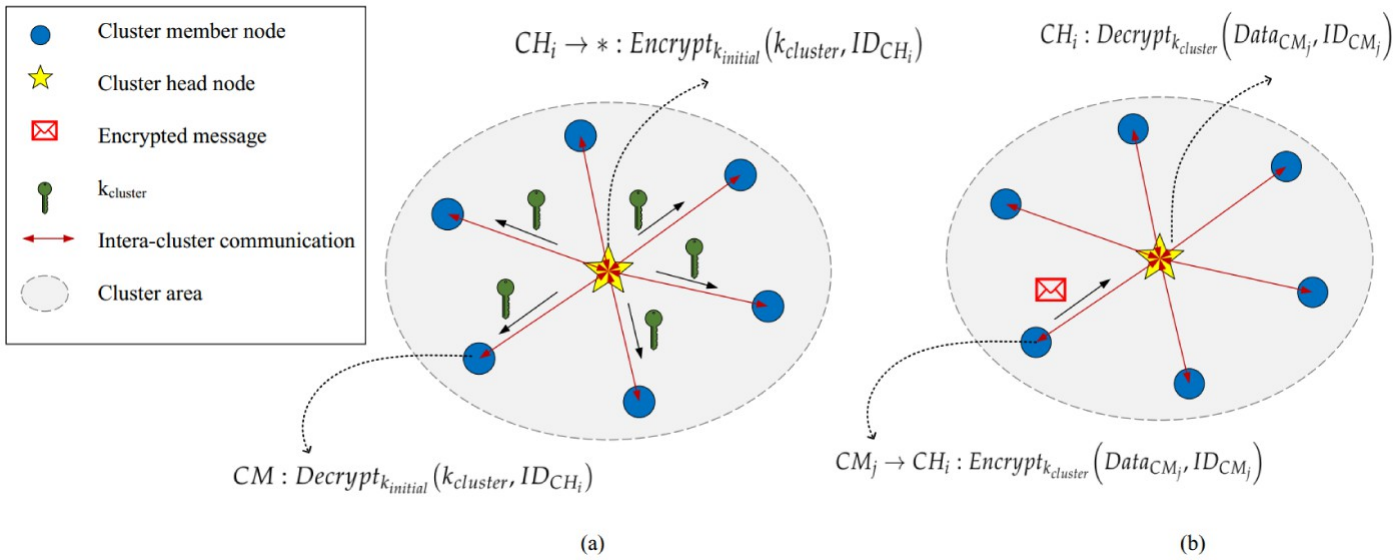
Intra-cluster security process.

This process is shown in Fig 3(a). Now, *CM*_*j*_ can perform the encryption process using *k*_*cluster*_ to secure its data (i.e. $Data_{CM_{j}}$). Then, *CM*_*j*_ forwards the encrypted data to *CH*_*i*_ according to Eq 22:

$$\begin{array}{c} CM_{j}\to CH_{i}:Encrypt_{k_{cluster}}(Data_{CM_{j}},ID_{CM_{j}}) \end{array}$$ (22)

When receiving the encrypted data packets of *CM*_*j*_, *CH*_*i*_ deciphers the encrypted packet. Then, it checks the identifier inserted into the packet and finally achieves $Data_{CM_{j}}$ based on Eq 23:

$$\begin{array}{c} CH_{i}:Decrypt_{k_{cluster}}(Data_{CM_{j}},ID_{CM_{j}}) \end{array}$$ (23)

Fig 3(b) shows the intra-cluster secure data transmission process. Moreover, Algorithm 2 describes the pseudocode of this process.

Now, the time complexity related to Algorithm 2 is analyzed. It includes an *IF* command (lines 5–9). This command consists of three commands (lines 6–8) with fixed execution times *t*_1_, *t*_2_, and *t*_3_, respectively. As a result, the time complexity related to Algorithm 2 is expresses with regard to Eq 24:

$$\begin{array}{c} T(n)=t_{1}+t_{2}+t_{3} \end{array}$$ (24)

By considering the fixed number *t* ≥ *t*_1_ + *t*_2_ + *t*_3_, in conclusion:

$$\begin{array}{c} T(n)=t_{1}+t_{2}+t_{3}\leq t \end{array}$$ (25)

Thus, the time complexity related to Algorithm 2 is obtained from Eq 26:

$$\begin{array}{c} T(n)\in O(1) \end{array}$$ (26)

**Algorithm 2** Intra-cluster security process

**Input**: *N*_*CM*_: Total number of CMs in the cluster *i*.

 *CH*_*i*_: Cluster head node in the cluster *i*.

 *CM*_*j*_: Cluster member nodes in the cluster *j*, where (*j* = 1, …, *N*_*CM*_).

**Output**: *k*_*cluster*_: Cluster key

 **Begin**

1: **CH**_**i**_: Produce *k*_*cluster*_;

2: **CH**_**i**_: Cipher *k*_*cluster*_ using *k*_*initial*_;

3: **CH**_**i**_: Send the encrypted *k*_*cluster*_ for all CMs in the cluster *i*;

4: **CM**_**j**_: Decipher the encrypted message and obtain *k*_*cluster*_;

5: **if** *CM*_*j*_ wants to securely send its data to *CH*_*i*_ **then**

6:  **CM**_**j**_: Cipher its data ($Data_{CM_{j}}$) using *k*_*cluster*_;

7:  **CM**_**j**_: Forward the encrypted data to *CH*_*i*_;

8:  **CH**_**i**_: Decipher the packet using *k*_*cluster*_ to achieve $Data_{CM_{j}}$;

9: **end if**

 **End**

#### 5.3.2 Inter-cluster security process

A safe connection between the cluster head nodes is guaranteed using asymmetric keys obtained from a elliptic curve cryptographic (ECC) method. It is a promising asymmetric key cryptosystem, which follows the theory of elliptic curves. The security of ECC depends on the difficulty of solving the elliptic curve logarithm problem. Although the deep explanation of this theory is out of the scope of this paper. For more details, refer to [52]. Hence, we select ECC in our scheme because if we consider a certain key size, ECC can provide better security than traditional cryptography systems such as the Rivest-Shamir-Adleman cryptosystem (RSA). For example, RSA guarantees its security with a price and large keys (for example, 1024 bits), and ECC can provide the same security level with smaller keys (for example, 160 bits). Asymmetric cryptosystems can provide better security levels in the network. CHs need higher security level because they have more communication overhead in the network. If these nodes are captured, the network performance faces more damage. Given what we said in the bootstrapping phase, before deploying nodes in the network, the sink node produces a pair of public-private keys (*k*_*pub*_−*k*_*pri*_) and stores them in the memory of CHs. These keys are used for securing messages of CHs. After deploying nodes in the network, CHs share their public key with each other.

If *CH*_*i*_ wants to securely communicate with *CH*_*j*_ to transfer $Data_{CH_{i}}$, it ciphers $Data_{CH_{i}}$ according to the public key of *CH*_*j*_ (i.e. $k_{pub_{j}}$). Eq 27 describes this process:

$$\begin{array}{c} CH_{i}\to CH_{j}:Encrypt_{k_{pub_{j}}}(Data_{CH_{i}},ID_{CH_{i}}) \end{array}$$ (27)

When receiving this message, *CH*_*j*_ deciphers the encrypted data by its private key ($k_{pri_{j}}$) to achieves $Data_{CH_{i}}$. Eq 28 presents this process.

$$\begin{array}{c} CH_{j}:Decrypt_{k_{pri_{j}}}(Data_{CH_{i}},ID_{CH_{i}}) \end{array}$$ (28)

**Fig 4:**
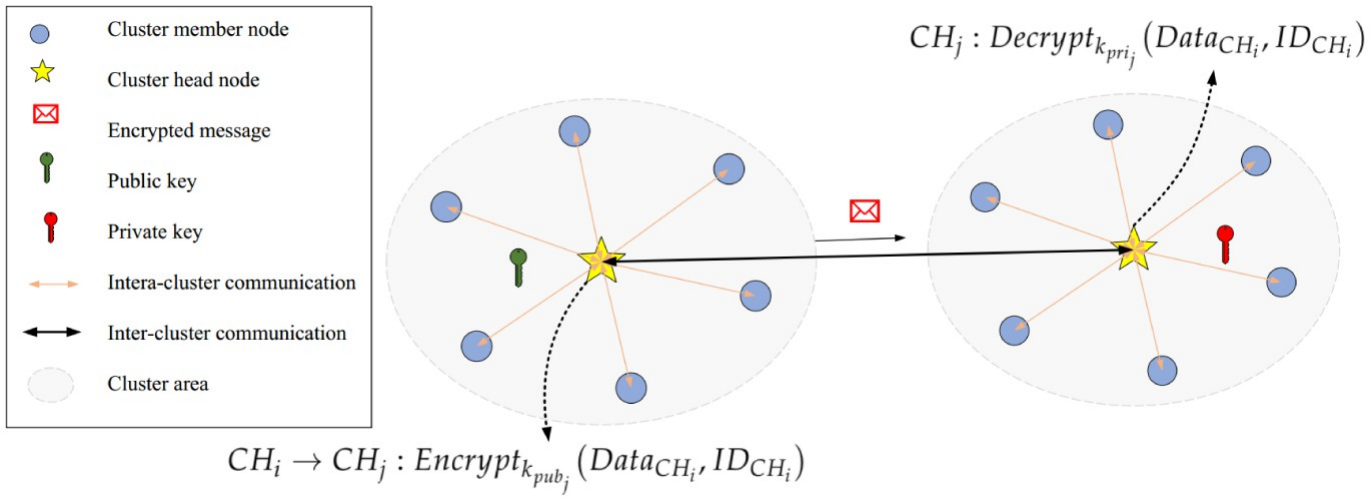
Inter-cluster security process.

Fig 4 shows the inter-cluster security process. Furthermore, Algorithm 3 illustrates the pseudocode related to the process.

Now, the time complexity corresponding to Algorithm 3 is discussed. In this algorithm, there is an *IF* command (lines 2–6), which includes three commands (3–5). These commands are executed at fixed times *c*_1_, *c*_2_, and *c*_3_, respectively. Therefore, the time complexity related to Algorithm 3 is calculated in accordance with Eq 29:

$$\begin{array}{c} T(n)=c_{1}+c_{2}+c_{3} \end{array}$$ (29)

Next, a fixed number *c* ≥ *c*_1_ + *c*_2_ + *c*_3_ is regarded:


$$\begin{array}{c} T(n)=c_{1}+c_{2}+c_{3}\leq c \end{array}$$ (30)

As a result, the time complexity related to Algorithm 3 is:

$$\begin{array}{c} T(n)\in O(1) \end{array}$$ (31)

**Algorithm 3** Inter-cluster security process

**Input**: *CH*_*i*_: Cluster head node *i*.

 *CH*_*j*_: Cluster head node *j*.

**Output**: A secure connection between *CH*_*i*_ and *CH*_*j*_.

 **Begin**

1: **CH**_**i**_: Share its public key ($k_{pub_{i}}$) with other CHs;

2: **if** *CH*_*i*_ wants to securely transfer its data to *CH*_*j*_ **then**

3:  **CH**_**i**_: Cipher $Data_{CH_{i}}$ using $k_{pub_{j}}$;

4:  **CH**_**i**_: Forward the encrypted data to *CH*_*j*_;

5:  **CH**_**j**_: Decipher the data by $k_{pri_{j}}$ and achieve $Data_{CH_{i}}$;

6: **end if**

 **End**

## 6 Security analysis

In this section, we study the security of our method in terms of data confidentiality, resistance to node capture attacks, eavesdropping attacks, and traffic analysis attacks.

- **Data confidentiality**: It is the most common security need because biosensors must protect their secret information against adversaries, which act maliciously to damage the confidentiality of their data. To prevent data leakage, data confidentiality is very essential. When capturing the biosensors, attackers endanger data confidentiality. Hence, health data transmission must be protected against eavesdroppers. To protect sensitive patient data, our scheme uses two encryption methods, namely RC4 and ECC so that the RC4 cryptosystem is employed for the intra-cluster security process, and the ECC technique is used to ensure security between CHs. In this case, an attacker cannot access the content of messages exchanged in the network because, in each cluster, biosensors encrypt their data using the cluster key before sending it to the CH. On the other hand, the distribution process of this cluster key is done in a secure manner because the relevant CH obtains this key from its key source and ciphers it by the initial key, and then sends this encrypted cluster key to CMs. Also, the communication links between CHs are secured using asymmetric keys. In inter-cluster communications, an attacker cannot access the content of messages exchanged between CHs because CHs only disseminate their public keys and maintain their private keys secretly. Thus, the attacker must capture all CHs to access their private keys. It is very difficult and time-consuming to capture all CHs. Therefore, our approach guarantees data confidentiality.
- **Resistance to node capture attacks**: In this attack, an adversary captures a biosensor node and carries out cryptanalysis to get secret data stored in its memory. After capturing this node, an attacker may insert fake data or extract health data, encryption keys, node ID, and routing information. In the proposed scheme, we check two modes:
  - **Compromising a cluster member node**: When an attacker compromises a cluster member node, then the information of this node such as its cluster key, the initial key, and the specific key of this node are disclosed. Therefore, this attacker accesses all data exchanged between CMs in the cluster because the adversary has the cluster key. However, as mentioned in Section 5.1, the sink node freshens the initial key periodically or when capturing a node in the network. Then, the sink node ciphers this new key by the specific key of each valid node and unicasts the encrypted key for valid nodes in the network. As a result, captured or dead nodes cannot access this key. Now, when the relevant CH updates the cluster key and sends this new key to its cluster members, the access of the attacker to the data of other nodes will be cut off because the cluster key is new. On the other hand, this attacker cannot access other clusters using the compromised cluster member. Hence, the access of this adversary is limited to the cluster related to this compromised node. This confirms that capturing a CM node does not have a negative effect on other clusters. Hence, the network can continue its normal performance.
  - **Capturing a cluster head**: If an attacker compromises a CH, then the secret information such as its ID, the cluster key, its private key, the public keys of other CHs, the initial key, and its specific key will be disclosed. However, other CHs can communicate with each other securely because this adversary only has their public keys, which are not secret. Hence, the attacker cannot extract the private keys of other CHs using this compromised CH. On the other hand, as mentioned in Section 5.1, the sink node freshens the initial key periodically or when capturing a node in the network. Then, the sink node ciphers this new key by the specific key of each valid node and unicasts it for valid nodes in the network. As a result, captured or dead nodes cannot access this key. Hence, the attacker cannot damage other clusters and decrypt their cluster key. However, CMs corresponding to the compromised CH cannot protect their data, and data packets in this cluster will be exposed. As a result, capturing this node has a local effect on the network.
- **Resistance to eavesdropping attacks**: This attack is an old security issue in which an eavesdropper sniffs the weakened links between biosensors in WBAN. By hearing the unsecured network path, this eavesdropper passively gets the data traffic (i.e. health data, routing information, node ID.). This attack is counteracted when network communication is secured using strong encryption keys. As mentioned above, the proposed scheme designs a secure key distribution process and a strong encryption operation, hence it can achieve confidentiality and avoid sniffing.
- **Resistance to Traffic Analysis**: This attack can threaten data confidentiality and privacy because the attacker checks and controls activities done by biosensor nodes to explore some information about the relevant node such as its role (i.e. CH or CM) and its position in the network. In the proposed scheme, this attack is counteracted because all data are encrypted and the key distribution process is also secure. Consequently, this attacker cannot obtain the content of packets because it does not have secret keys in the network. As mentioned earlier, cluster members employ a symmetric encryption technique to guarantee data confidentiality within a cluster. Also, our scheme can protect CHs using asymmetric keys because the adversary cannot achieve the private keys of all CHs to decrypt their data packets.

## 7 Performance analysis

In this section, we evaluate routing overhead in different routing method, including the proposed scheme, SecAODV [40], SMEER [30] and LEACH-C [36] because communication overhead is the most important factor affecting energy consumption. This factor is equal to the number of control messages exchanged in the network when calculating various paths. As a result, a successful routing protocol must manage routing overhead to optimize network performance in terms of energy consumption. To compare routing overhead in different routing protocols, we assume that *N* nodes are available in the network. They include *N*_*CH*_ cluster head nodes and *N*_*CM*_ cluster member nodes. Also, it is assumed that each node has maximum *N*_*neighbor*_ neighboring nodes in the network.

In our scheme, each node transmits a hello message including its location and energy level to its neighboring nodes and receives *N*_*neighbor*_ hello messages from them. In general, the total number of sent/received hello messages in each node equals 1 + *N*_*neighbor*_. Then, the proposed scheme uses LEACH [44] to cluster network nodes. According to LEACH, cluster head nodes are selected in a random manner. Then, each CH disseminates an advertisement message to announce itself as CH. In the worst case, each non-CH node receives *N*_*CH*_ advertisement messages from CHs in the network. In this case, it joins the CH, which includes the maximum signal strength, and transmits a join-request message to the CH. In this step, the CH receives *N*_*CM*_ join-request messages from its CMs. In the routing process, each CM forwards its data to the CH using a single-hop manner. To establish paths between CHs, each cluster head node applies LCA to prioritize the neighboring CHs. Then, this CH sends the route request (RREQ) message to the half of neighboring CHs with higher priorities. This routing process is similar to AODV, and its routing overhead is equal to *N*_*CH*_ message in each CH node. In the security phase, each CH sends a message including the cluster key to its cluster member nodes, shares its public key with other CHs, and receives *N*_*CH*_ messages including their public key from other CHs. Based on the mentioned points, we can state that:

The routing overhead in CHs is equal to: (1 + *N*_*neighbor*_) + (1 + *N*_*CM*_) + 2(1 + *N*_*CH*_)

The routing overhead in CMs is equal to: (1 + *N*_*neighbor*_) + (1 + *N*_*CH*_) + 1

SecAODV employs uses LEACH to divide network nodes in different clusters. As mentioned above, LEACH chooses cluster heads randomly. Next, the chosen CH propagates an advertisement message to announce itself as CH. In the worst case, each non-CH node receives *N*_*CH*_ advertisement messages from CHs. In this case, it joins the CH, which includes the maximum signal strength, and transmits a join-request message to the CH. In this step, the CH receives *N*_*CM*_ join-request messages from its CMs. In the routing process, each CM transfers its data to the relevant CH using a single-hop manner. To establish paths between CHs, SecAODV uses an AODV-based routing method. Then, this CH sends the route request (RREQ) message to its neighboring CHs. Therefore, the routing overhead of this process is equal to *N*_*CH*_ message in each CH node. In the security phase, each CH sends its cluster key to its CMs, transmits its public key to other CHs, and receives *N*_*CH*_ messages including their public key from other CHs. Based on the mentioned points, we can state that:

The routing overhead in CHs is equal to: (1 + *N*_*CM*_) + 2(1 + *N*_*CH*_)

The routing overhead in CMs is equal to: (1 + *N*_*CH*_) + 1

In SMEER, all nodes transfers their location information to the sink node. Then, the sink node is responsible for preforming the clustering operation using K-means and selecting CHs based on the ALO algorithm. Finally, BS sends the CH information to network nodes (*N*_*CH*_ messages). Next, CHs disseminate an advertisement message to other nodes to announce themselves as CHs. In the worst case, non-CH nodes receive *N*_*CH*_ messages from different CHs. They join the high-quality CH and send a join-request message to the CH. In this case, CHs receive *N*_*CH*_ join-request messages from the cluster member nodes. In the next step, each node calculates its private-public keys and shares its public key with other nodes in the network. Also, it receives *N* messages including public keys from other nodes. In the last step, CHs exchange information about angular position, angle, and energy with each other to find the best next-hop node using a spherical manner. Thus, the routing overhead in SMEER is as follows:

The routing overhead in CHs: (1 + *N*_*CH*_) + (1 + *N*_*CM*_) + (1 + *N*_*CH*_ + *N*_*CM*_) + (1 + *N*_*neighbor*_)

The routing overhead in CMs: (1 + 2*N*_*CH*_) + (1 + *N*_*CM*_) + (1 + *N*_*CH*_ + *N*_*CM*_)

In LEACH-C, all nodes transmits their position and energy level to the BS in a single-hop manner. Then, BS applies the simulated annealing algorithm to pick out CHs. Then, BS sends the CH information to the network nodes. Then, CH broadcasts an advertisement message to other nodes. Non-CH nodes receive *N*_*CH*_ advertisement messages from CHs in the worst case. They join the nearest CH. Next, CMs send a join-request message to the CH, meaning that the CH receives *N*_*CM*_ join-request messages from its CMs. In this case, the routing overhead is stated as follows:

Routing overhead in CHS: (1 + *N*_*CH*_) + (1 + *N*_*CM*_)

Routing overhead in CMs: (1 + 2*N*_*CH*_)

Table 2 compares the routing overhead in various approaches. Based on this table, we found that LEACH-C has a lower routing overhead than our scheme and SMEER because LEACH-C presents any security mechanism.

**Table 2:** Comparison of different approaches in terms of routing overhead.

Scheme	Routing overhead in CHs	Routing overhead in CMs
Proposed	(1 + *N*_*neighbor*_) + (1 + *N*_*CM*_) + 2(1 + *N*_*CH*_)	(1 + *N*_*neighbor*_) + (1 + *N*_*CH*_) + 1
SecAODV	(1 + *N*_*CM*_) + 2(1 + *N*_*CH*_)	(1 + *N*_*CH*_) + 1
SMEER	(1 + *N*_*CH*_) + (1 + *N*_*CM*_) + (1 + *N*_*CH*_ + *N*_*CM*_) + (1 + *N*_*neighbor*_)	(1 + 2*N*_*CH*_) + (1 + *N*_*CM*_) + (1 + *N*_*CH*_ + *N*_*CM*_)
LEACH-C	(1 + *N*_*CH*_) + (1 + *N*_*CM*_)	(1 + 2*N*_*CH*_)

## 8 Simulation and evaluation of results

In this section, our scheme is evaluated and simulated using the network simulator version 2 (NS2). For simulating our scheme, the network involves 100 sensor nodes. These nodes are fixed. Also, the size of network is equal to 50 × 2500*m*^2^. The position of the sink node is in the middle of network. The packet size is 1024 bits. Normal nodes have 0.5*J* energy, and the energy of CHs is equal to 1*J*. Additionally, the simulation time is regarded 30 seconds. To enhance the accuracy of evaluation results, the simulation operation is repeated 25 times. Other factors related to the simulation operation are summarized in Table 3. Our approach is analyzed with regard to end-to-end delay, throughput, consumed energy, packet delivery ratio (PDR), and packet loss rate (PLR) and the results are presented in comparison with SecAODV [40], SMEER [30], and LEACH-C [36].

**Table 3:** Simulation parameters.

Parameter	Value
Simulation software	NS2
Network dimensions	50 × 2500*m*^2^
Location of Sink	In the middle of network
All number of nodes	100
Primary energy of CHs	1 J
Primary energy of normal nodes	0.5 J
Antenna	Omni-Antenna
Packet size	1024 bit
Mac protocol	IEEE 802.11
Simulation time	30 s

### 8.1 End-to-end delay

**Fig 5:**
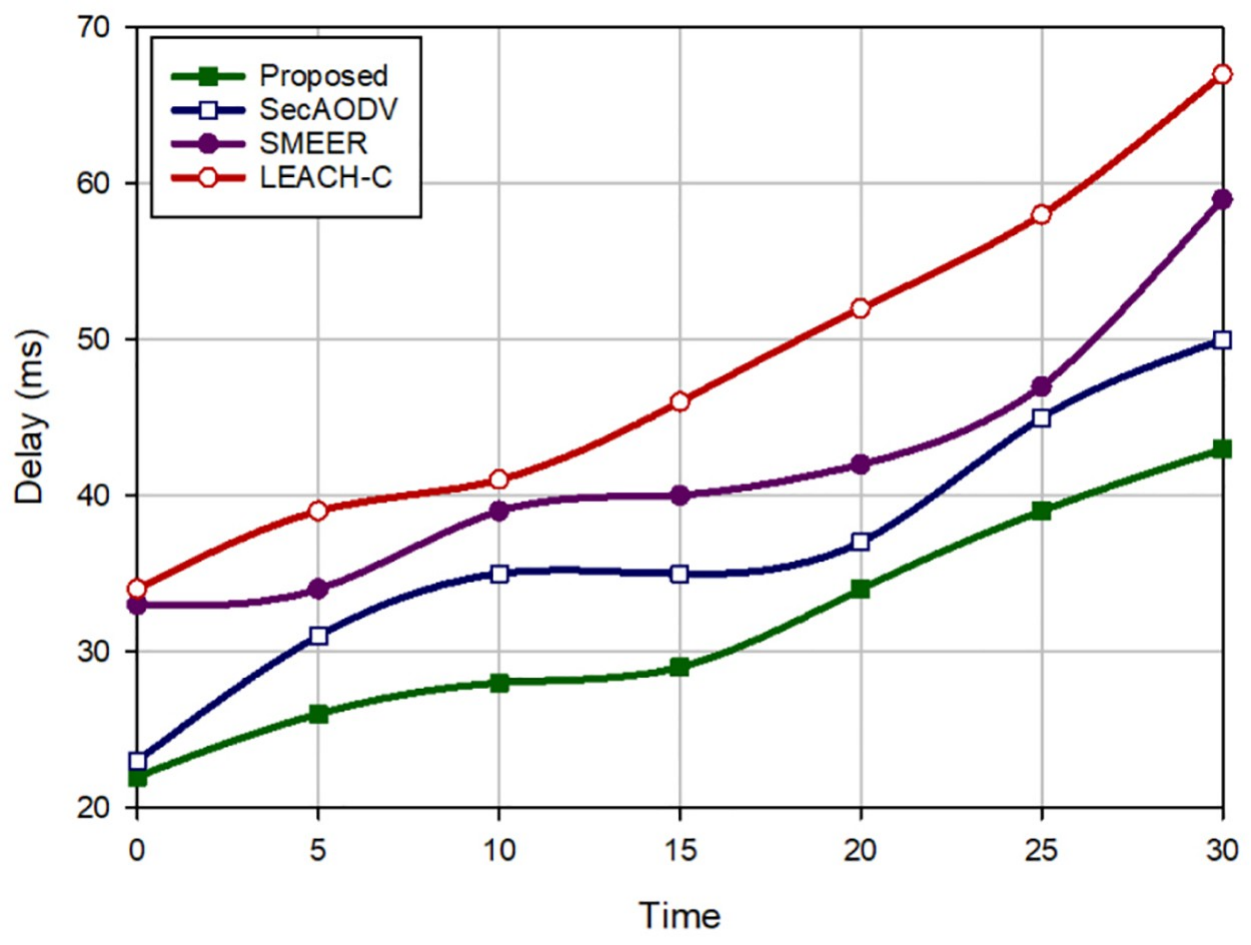
Delay in different methods.

End-to-end delay means the total time required to deliver packets to the sink node. Fig 5 expresses a comparison of delay in different routing approaches. According to this figure, our method experiences the minimum delay and reduces this factor by 13.67%, 24.83%, and 34.42% in comparison with SecAODV, SMEER, and LEACH-C, respectively. Therefore, our scheme is faster than other schemes in the data transmission process while the routing process in SecAODV requires more time compared to that in our scheme. Also, SMEER focuses only on asymmetric encryption method to provide secure channels. This increases delay when transferring data in SMEER. While our scheme and SecAODV design a hybrid cryptography method so that they utilize the symmetric key cryptography for securing the communication links between CMs. In each cluster, CMs use cluster key for encrypting their data. On the other hand, our scheme and SecAODV use an asymmetric cryptosystem to secure connections between CHs in the network. Other point is to SMEER designs a clustering algorithm using K-means and the ALO algorithm. This process has high computational overhead, which leads to high delay in the network. Additionally, the performance of LEACH-C depends on the simulated annealing algorithm, which causes a high computational overhead and a long delay in the network. In contrast, our scheme and SecAODV divide sensor nodes in different clusters using the LEACH algorithm, which is faster than LEACH-C and SMEER. The third point is that our scheme takes into account connection quality and residual energy when discovering new routes. These parameters lead to the creation of more stable routes compared to SMEER and LEACH-C. This advantage lowers route failure, which leads to low delay in the routing process.

### 8.2 Throughput

**Fig 6:**
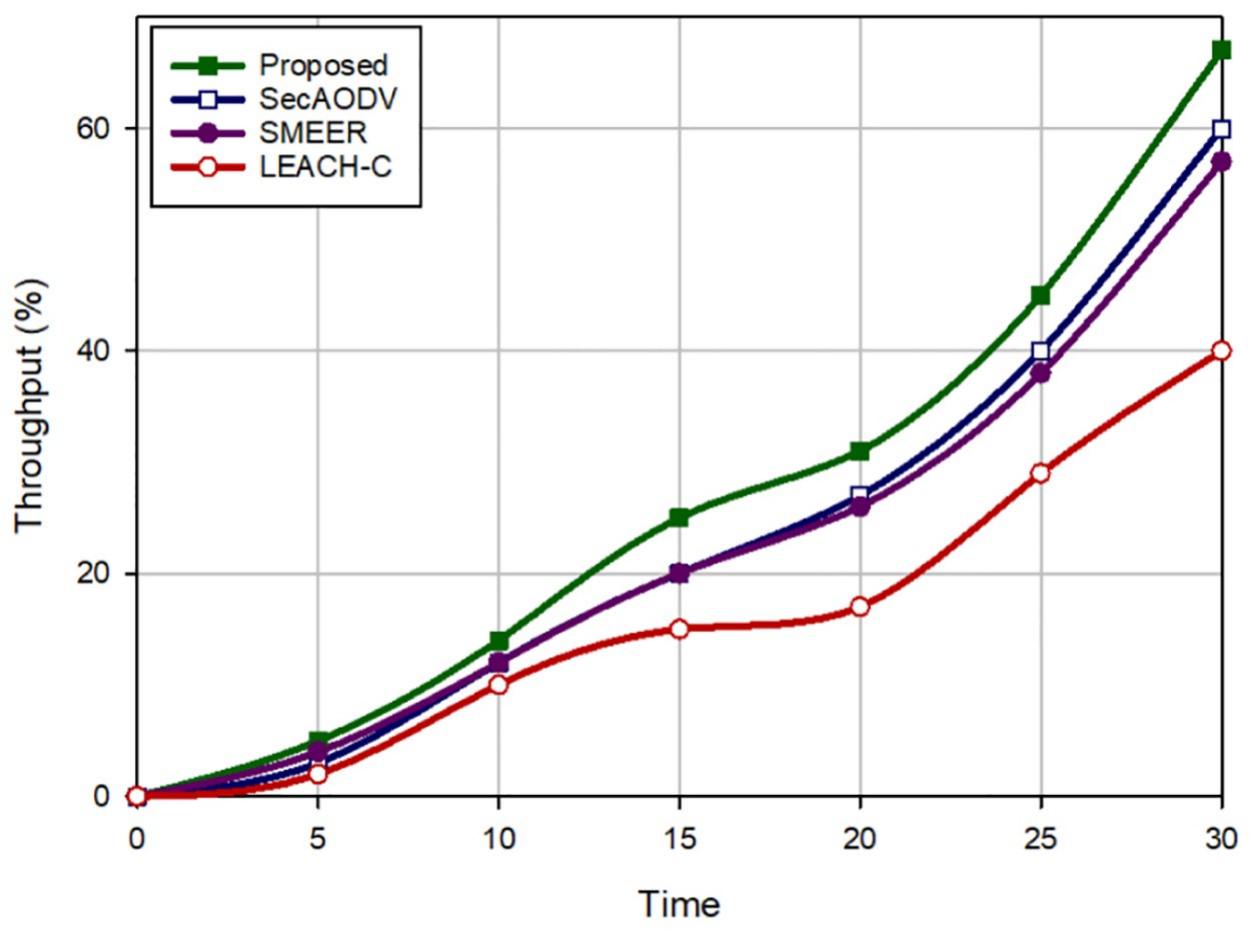
Throughput in different methods.

Throughput means the ratio of packets received by the receiver node to latency needed for transmitting the packets. Fig 6 presents a comparison of throughput in different protocols. According to this figure, our scheme has the highest throughput and enhances this parameter by 15.44%, 19.11%, and 65.53% in comparison with SecAODV, SMEER, and LEACH-C, respectively. Its reason is that our scheme decreases delay compared to other methods. This issue is mentioned in Section 8.1. Moreover, our scheme uses high-energy nodes as the next-hop nodes and considers link quality when creating routes. Thus, our scheme improves the data transfer process, which leads to higher throughput than others.

### 8.3 Energy consumption

**Fig 7:**
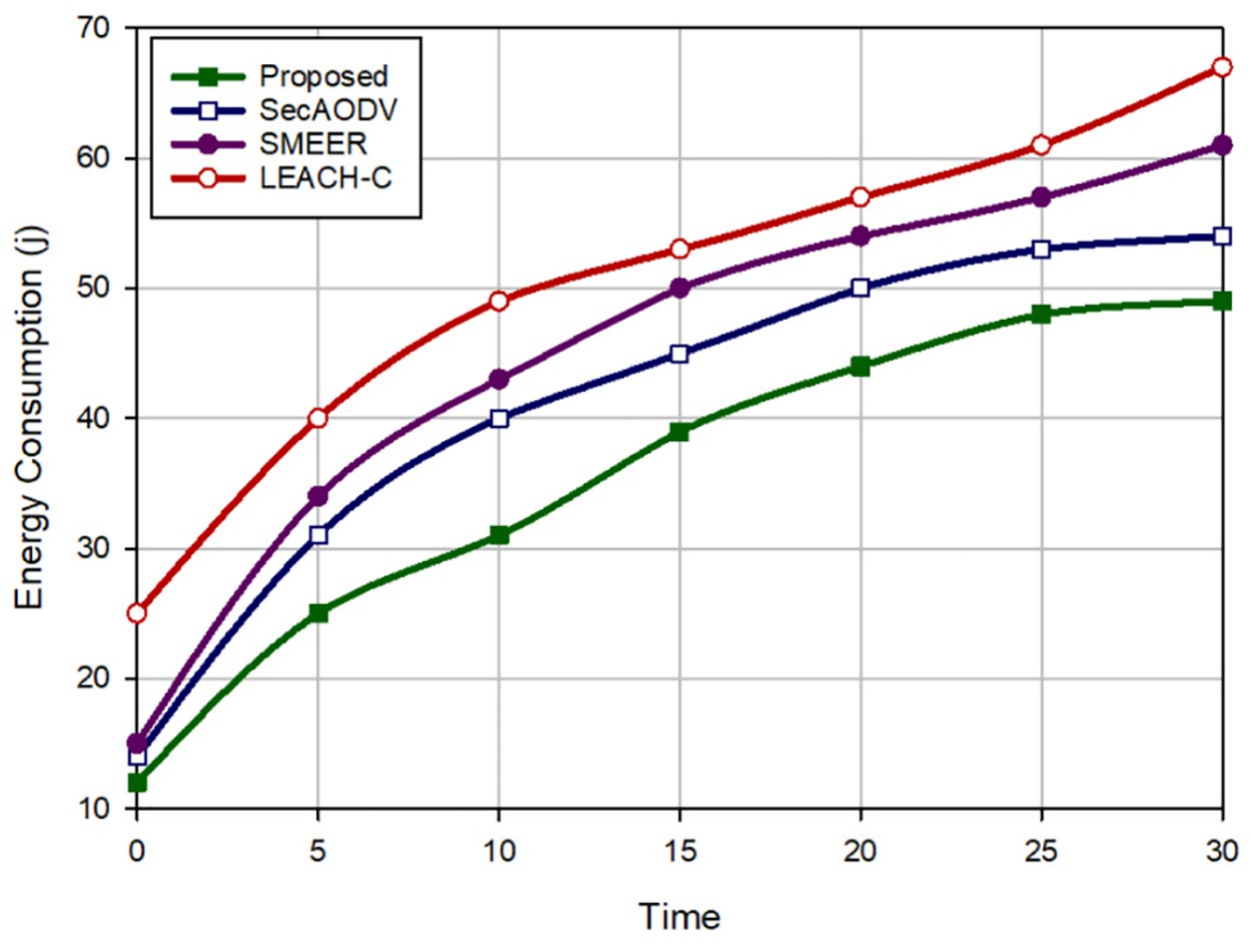
Energy consumption in different methods.

Routing methods are compared regarding energy consumption in Fig 7. Our scheme lowers the consumed energy and decreases this parameter by 13.58%, 21.02%, and 29.53% in comparison with SecAODV, SMEER, and LEACH-C, respectively. This is because LEACH-C defines the communication between CHs and the sink node as a single-hop manner, which leads to high energy consumption. SMEER utilizes a multi-hop manner when transferring packets to the sink node. It has better performance than LEACH-C. However, SMEER selects the next-hop node in accordance with distance and the angle between nodes. While more appropriate parameters, especially energy, can be considered to lower energy consumption. SecAODV creates multi-hop paths between CHs and BS and takes into account remaining energy, distance, connection quality, and hop count in the routing process. Our scheme utilizes a multi-hop route between CHs and the sink node and considers important parameters, especially energy and connection quality, in the routing operation. Thus, our scheme can form stable paths and reduce route failure, which leads to low energy consumption in the data transmission process.

### 8.4 Packet delivery rate (PDR) and packet loss rate (PLR)

**Fig 8:**
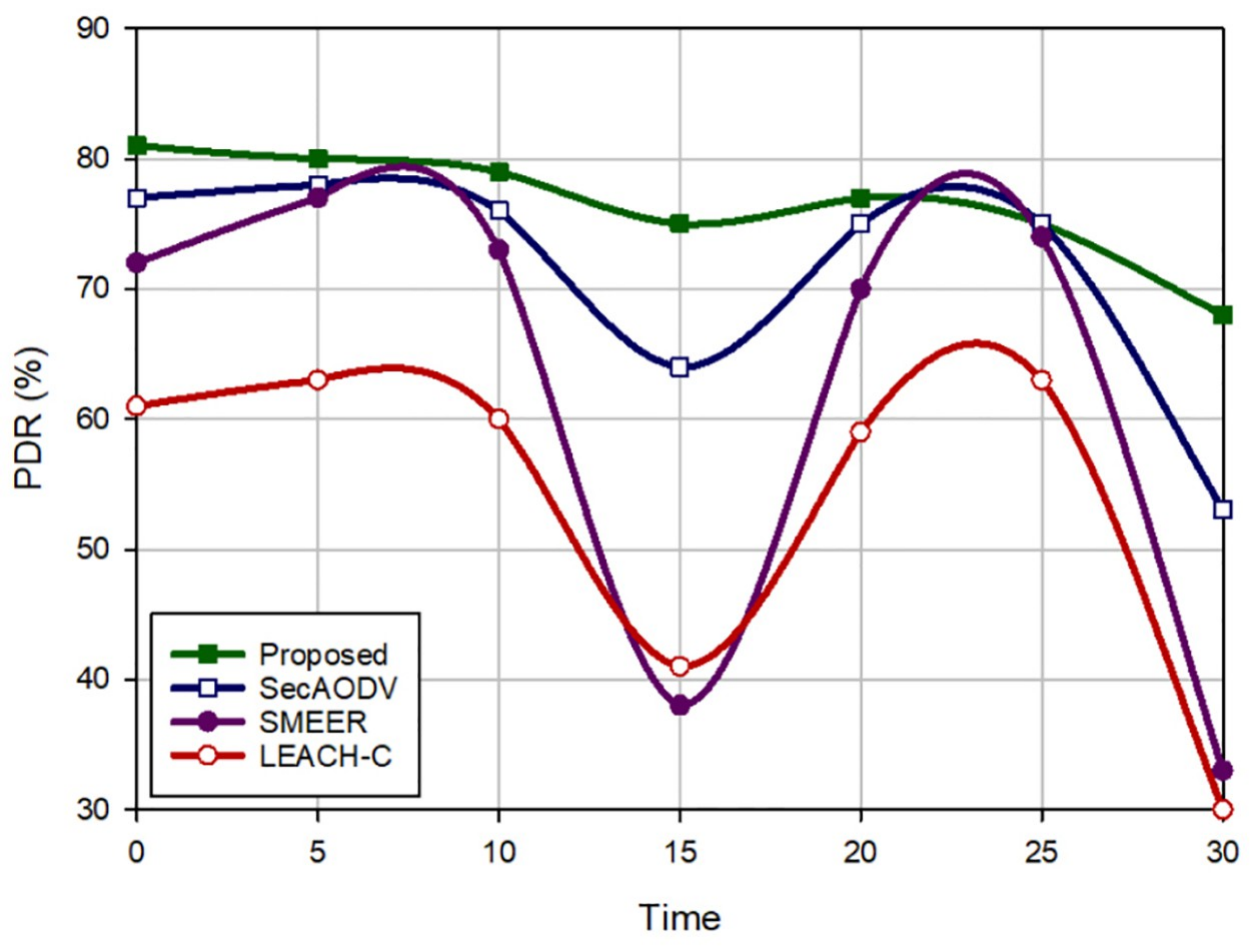
Packet delivery rate in different methods.

**Fig 9:**
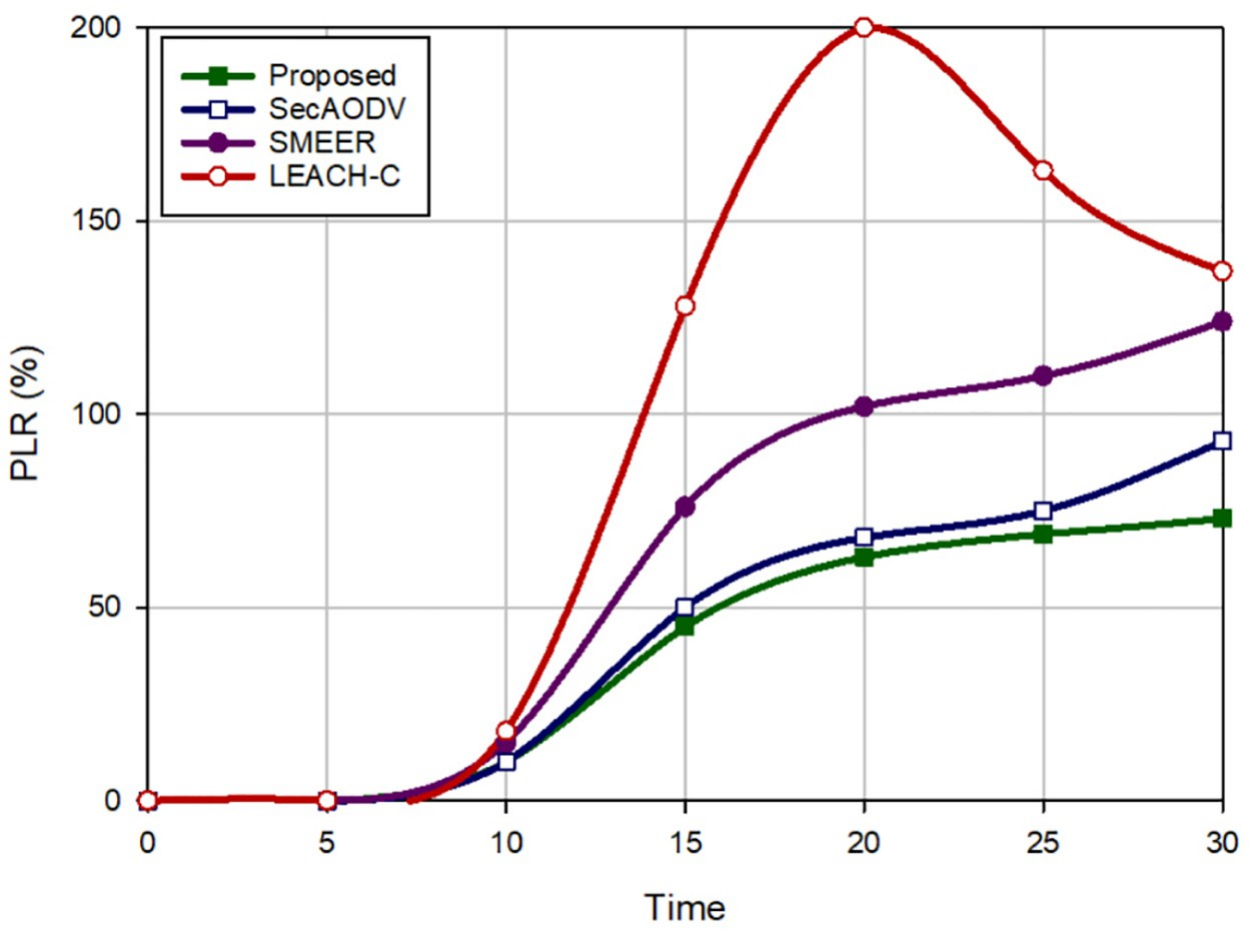
Packet loss rate in different methods.

Packet delivery rate is equal to the ratio of the data packets received by the receiver to the total number of packets. Fig 8 expresses a comparison of PDR in different approaches. According to this figure, our scheme maximizes PDR and enhances this parameter by 7.44%, 22.43%, and 41.90% in comparison with SecAODV, SMEER, and LEACH-C, respectively. Also, packet loss rate is equal to the ratio of lost data packets to the total number of packets. Fig 9 presents a comparison of PLR in different approaches. Based on this figure, our scheme lowers PLR by 12.16%, 39.11%, and 59.75% in comparison with SecAODV, SMEER, and LEACH-C, respectively. This is because our proposed method has a good performance in terms of delay and throughput. LEACH-C has the weakest performance in terms of PDR because in this scheme, CHs has high routing overhead and high energy consumption. This is because they create a single-hop connection to the sink node. This decreases the packet delivery rate in LEACH-C. Also, SMEER does not regard energy and connection quality when forming routs. Thus, it may establish unstable routes, which leads to packet loss. Our scheme considers these parameters when finding routes, and creates more stable paths, which leads to higher PDR than others. Also, SecAODV regards energy and connection quality in the routing process and forms stable paths, consequently, it has a high PDR.

## 9 Conclusion

In this paper, we proposed a secure routing approach using the league championship algorithm (LCA) for WBSN. This method involves two main parts. In the first part, the cluster head nodes use LCA to select the best next-hop node. Moreover, a fitness function was presented based on distance, energy and link quality. In the second step, a lightweight security mechanism was presented. In this step, the communication links between the CHs and cluster members were secured using symmetric encryption technique. Ultimately, the communication links between CHs was also secured by the ECC cryptography. Then, the proposed method was simulated using NS2 and its results were analyzed with regard to latency, throughput, consumed energy, packet delivery ratio, and packet loss ratio in comparison with SecAODV, SMEER and LEACH-C. These results show a successful performance of the proposed method, especially in reducing energy consumption than other routing methods. In future research directions, we compare our proposed scheme with newest secure routing methods and consider a real-world implementation to validate the performance of our scheme. Also, we will use new techniques such as fuzzy logic, meta-heuristic algorithms, and machine learning technique to design strong security mechanisms in future.

## Supporting information

S1 Text (TXT)

S2 Text (BST)
